# The “unclear problem” category: an analysis of its patient and dispatch characteristics and its trend over time

**DOI:** 10.1186/s12873-022-00597-6

**Published:** 2022-03-12

**Authors:** Sterre Otten, Cassandra Rehbock, Thomas Krafft, Martin Vang Haugaard, Eva Pilot, Stig Nikolaj Blomberg, Helle Collatz Christensen

**Affiliations:** 1 Department of Health, Ethics and Society, Faculty of Health, Medicine and Life Sciences (FHML), Care and Public Health Research Institute (CAPHRI), Maastricht University, Maastricht, The Netherlands; 2 Copenhagen Emergency Medical Services, Ballerup, Denmark; 3 Danish Clinical Quality Program (RKKP), Frederiksberg Hospital, Frederiksberg, Denmark

**Keywords:** Emergency medical services, Dispatch centers, Unclear problem, Category, Copenhagen

## Abstract

**Background:**

An effective emergency medical dispatch process is vital to provide appropriate prehospital care to patients. It increases patient safety and ensures the sustainable use of medical resources. Although Copenhagen has a sophisticated emergency medical services (EMS) system with a significant focus on public welfare, more than 10% of emergency cases are still being categorized as an "unclear problem category" (UPC) and are thus not categorized as "symptom-specific". Therefore, the objective of this research is to gain a better understanding of the patient and dispatch characteristics of emergency cases categorized as "unclear".

**Methods:**

This register-based study based on medical emergency cases data describes patient and dispatch characteristics of emergency cases categorized as “unclear” through the use of numbers and proportions. Moreover, these cases were compared to non UPC cases. Use of UPC was stratified by month to determine the impact of alerting medical dispatchers to reduce its use.

**Results:**

From 296,398 included cases UPC accounted for 11.4% of the cases. The median age of those triaged with the UPC was 66 years vs 58 years for individuals triaged with other symptom-specific categories.

Moreover, after having been triaged with the UPC, 9,661 (34.7%) of the dispatched EMS vehicles ended up being cancelled. Sensitizing medical dispatchers about the use of the UPC likely contributed to the decreased use of the UPC over time.

**Conclusion:**

The UPC has different dispatch characteristics than the symptom-specific categories, with potential negative effects on the medical dispatch process. Moreover, the median age of individuals triaged with the UPC is higher than those triaged with symptom-specific categories. Nonetheless, the use of the UPC decreased throughout the study period after the medical dispatchers were alerted about the implications of its use.

## Background

The World Health Organization identifies emergency medical services (EMS) as an integral part of any effective and functional healthcare system. The emergency medical dispatch center (EMDC) is the first point of contact in the case of a life-threatening or medical emergency [1]. A prompt prehospital response is conducive to a better prognosis of patients and timely access to EMS [2]. Throughout Denmark, all emergency or 1–1-2 calls are first answered by the police, except in the Capital Region of Denmark, where the Copenhagen fire brigade answers these calls. The call-taker assesses the situation according to the information provided via the call and locates the incident's site. As of 2011, a call of medical nature is then forwarded to a regional EMDC [3, 4].

While identifying a specific complaint is crucial, the Unclear Problem Catagory (UPC) remains a frequent category used by dispatchers to classify emergencies. The categorization of an emergency as UPC discloses that the medical dispatcher cannot determine the exact medical cause of the case in question. Nonetheless, an assessment of the level of urgency is still performed based on the description of the caller [5, 6].

On the one hand, using the UPC could cause delayed responses to other life-threatening incidents with a potentially higher level of urgency [7]. On the other hand, it could lead to the use of a higher emergency priority level than necessary, resulting in the inefficient use of medical resources. Medical dispatch accuracy is vital in optimizing the balance between patient needs and available prehospital resources [5].

Since 2015, medical dispatchers at the EMS in Copenhagen have been made more aware of the use of the UPC in an attempt to reduce the use of the UPC in the categorization process. UPC was addressed through staff meetings focused on the topic of UPC. It is relevant to investigate whether this sensitization has had an impact on the use of the UPC in medical dispatching at the EMS in Copenhagen.

Although Copenhagen already has a sophisticated emergency care system with a significant focus on public welfare [4], more than 10% of emergency cases remain categorized as UPC and further improvements in the system can thus be made. Considering the potentially harmful implications of using the UPC, its investigation is needed to improve the dispatching process further. The aim of this study was to investigate the patients and dispatch characteristics of UPC cases, as well as explore the use of the UPC over time.

## Methods

### Study design

A register-based study based on emergency medical cases from the EMDC in Copenhagen was conducted. The research was done according to the framework of the EMDC Copenhagen, Denmark's quality assurance protocol. The project was approved by the executive level of the EMDC and Maastricht University (FMHL/BEPH/2020.016). The study period entailed data from January 1^st^, 2017, until December 31^st^, 2019. The main research population was identified based on the emergency cases categorized as UPC. The control group was based on cases based on the other 37 symptom-specific Danish Index categories, gathered over the same time period. Cases with no dispatch criteria were excluded.

### Categorization in emergency medical dispatching

At EMDC Copenhagen, healthcare professionals, either nurses or paramedics, answer the calls [8]. They must then assess the situation and choose a response using the criteria-based emergency medical dispatch (CBD) system, also referred to as the Danish Index for Emergency Care. The Danish Index, used by all regions in Denmark [3], consists of 38 criteria, including the "unclear problem" category (UPC), which is supposed to be used very conservatively (2–5% of cases). The various criteria correspond to clinical signs, symptoms or incidents and it aids the professional to decide the response based on the implicated level of urgency [5].

The CBD categorization using the Danish Index is the first step in the emergency medical dispatching process. It leads to more specific questions that enable the medical dispatcher to initiate the appropriate and corresponding priority level (ranging from A-E). Level A includes life threatening/potentially life-threatening symptoms; B includes urgent, yet not life-threatening symptoms; C is for non-urgent conditions that still require an ambulance; D has non-urgent cases requiring supine patient transport; and E is for cases that merely require medical advice [5]. Simultaneously, a red response (an immediate response with lights and sirens), an orange response (an immediate response without lights and sirens), a yellow response (a non-urgent response with the needed resources available), a green response (non-urgent) and a blue response (merely medical advice, for instance referring the patient to their general practitioner) can be dispatched [5]. Upon arrival at the scene, the paramedics can revise the category and either upgrade or downgrade the urgency of transport to the hospital. Similarly, they can revise the criteria if they see fit, so the appropriate criteria are relayed to the receiving hospital.

### Data analysis

Data was stratified upon UPC and non-UPC. Age was grouped (0–17 years, 18–65 years and 65 + years) and comparisons were calculated with medians, IQR, number and percentages. Comparisons were made to patients that were allocated to the remaining 37 symptom-specific categories.

Most frequent response, numbers of vehicles dispatched and number of cancelled transports were described using numbers and percentages. Moreover, the usage of the UPC over time was explored, to investigate the effect after making medical dispatchers more aware of the implications related to the use of the UPC.

## Results

**Fig. 1:**
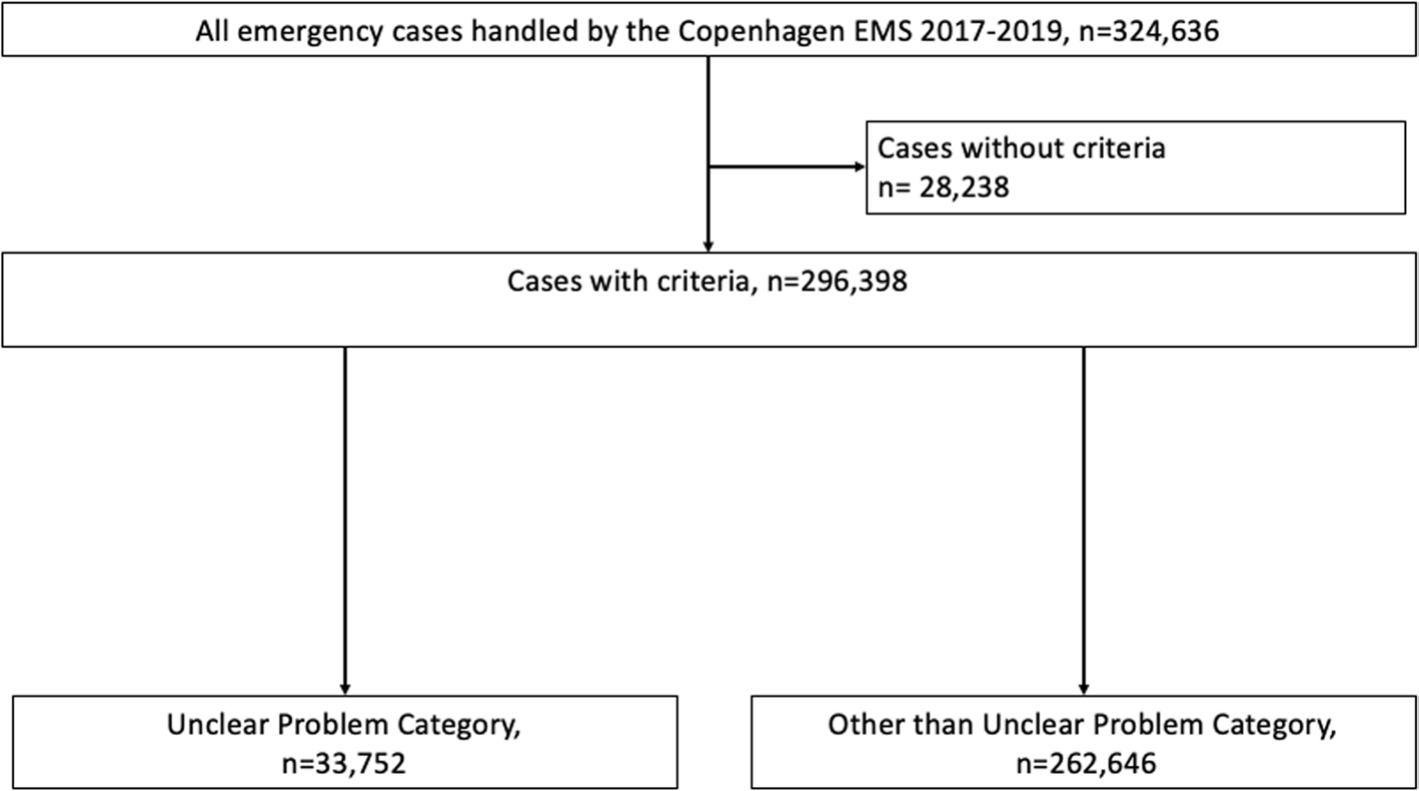
Flowchart demonstrating the inclusion criteria of the cases included in this study

From 2017 to 2019 the EMDC Copenhagen responded to 324,536 emergency cases (Fig. 1). We excluded 28,838 (9%) of the cases dispatch criteria were missing. After exclusions 33,752(11.4%) cases registered with UPC was identified.

Table 1 (medical dispatch information based on non UPC cases & UPC cases specifically) illustrates sociodemographic and medical dispatch information of non UPC cases and UPC cases over the same study period, from 01–01-2017 to 31–12-2019.

**Table 1:** Sociodemographic and medical dispatch information of non UPC cases & UPC cases specifically registered at EMDC Copenhagen, Denmark. B = a car is dispatched within an hour F = no response sent out, A = high urgency level

	Non UPC cases	UPC cases specifically
Total number of cases	262,646	33,752
The MEDIAN age (Years, median and IQR)	58 (42)	66 (32)
0–17 years	21,325 (8.1%)	958 (2.8%)
18–65 years	111,445 (42.4%)	11,623 (34.4%)
65 + years	95,773 (36.5%)	13,600 (40.3%)
Missing	34,103 (13.0%)	7,571 (22.4%)
Number of cases 2017	86,172 (32.8%)	12,743 (37.7%)
Number of cases 2018	88,391(33.5%)	12,261 (36.3%)
Number of cases 2019	88,083 (33.5%)	8,748 (25.9%)
Most frequent responses		
A	118,945(45.3%)	10,672(31.6%)
B	98,432(37.5%)	11,626(34.4%)
F	28,800(11.0%)	9,942(29.5%)
Number of emergency medical vehicles dispatched^a^		
0	37,659(14.3%)	10,250(30.4%)
1	176,079(67.0%)	19,715(58.4%)
2	82,566(31.4%)	3,290(9.7%)
EMS vehicles dispatched that were subsequently cancelled	84,099 (29.7%)	9,661 (34.7%)
Reasons for EMS vehicle cancellation		
Patient does not want to be brought in	24,981(9.5%)	3,688 (11.0%)
Vehicle was reassigned for other incident	17,159(6.5%)	1,900(5.6%)
Cancelled by ambulance on scene	6,290(2.4%)	370 (1.1%)
Other, Dispatch center order	4191 (1.6%)	718 (2.1%)
Criteria changed by paramedics on scene	24,426 ( 9.3%)	4,050 ( 12.0%)
Response changed by paramedics or dispatchers	42,023 (16.0%)	4,725 (14.0%)

^a^ '0 vehicles dispatched' covers cases where the response 'F' was assigned or cases where the response given is that the patient is asked to self-transport to the emergency department

This table demonstrates some key trends about non UPC cases as well as UPC cases specifically. Some notions that stand out from this table are that patients triaged with non UPC categories have a younger median age than those triaged with the UPC. Moreover, the number of vehicles cancelled is lower for all non UPC cases than for those triaged with the UPC. Lastly, the number of non UPC cases has shown a fairly consistent trend over the years while the number of UPC cases has decreased.

**Fig. 2:**
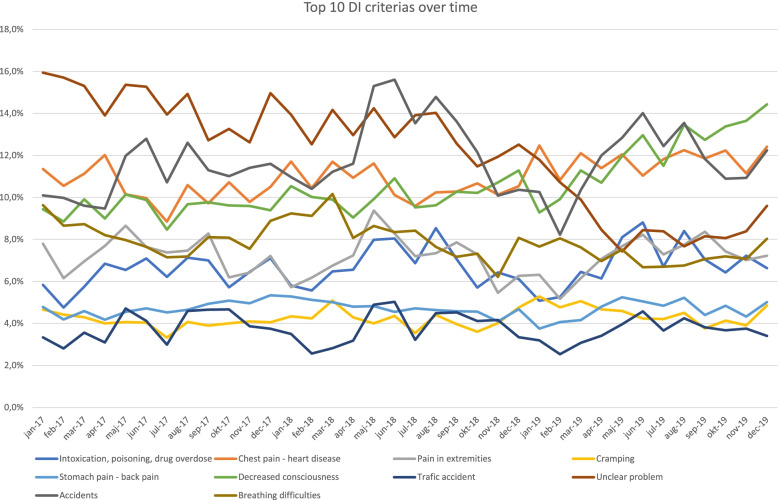
Line chart demonstrating the top 10 dispatch criteria used over time at the EMDC from January 1^st^, 2017 until December 31^st^, 2019. Data source: system data from the emergency medical dispatch Center in Copenhagen, 2019

The median age of the patients triaged with the UPC was 66 years. After having been triaged with the UPC, 9,661 (34.7%) of the EMS vehicles ended up being cancelled. The graph in Fig. 2 below depicts the top dispatch criteria reported at the EMDC in Copenhagen during the study period. It can be seen that at the beginning of 2017, the most used dispatch criteria used by medical dispatchers were related to "unclear" cases. However, as can be seen in Table 1 as well as in Fig. 2, the trend is visibly declining and faster than any other symptom-specific dispatch criteria.

## Discussion

This study aimed to create a structured overview of the patient and dispatch characteristics of UPC cases, as well as to investigate the effects of sensitizing medical dispatchers to reduce the use of the UPC. The research demonstrated that the median age of patients triaged with the UPC was higher than the age of patients triaged with symptom-specific categories. Moreover, the UPC's use has been declining over the years, demonstrating a possible effect of alerting medical dispatchers about the implications of using the UPC. On top of that, EMS vehicles dispatched with the UPC were more often cancelled than EMS vehicles dispatched with the symptom-specific categories.

### Sociodemographic determinants related to the use of the "unclear problem" category

The median age of patients triaged with the UPC was higher than the age of the patients triaged with the symptom-specific categories (66 vs. 58 years). In line with these results, a study based on emergency medical patients in hospitals in Denmark and California has demonstrated that non-specific diagnoses such as "other symptoms" and "other factors" constituted large groups in the elderly patient population [9]. Although this study considered the patient population in hospitals, it correlates with the higher median age of patients triaged with the UPC.

The fact that the median age of patients triaged with the UPC is higher than the median age of patients triaged with symptom-specific categories may be explained by the elderly’s reduced capability to exchange information in a clear and precise way [5]. Moreover, elderly patients often present with more difficult problems. Namely, a study conducted by Wachelder et al. [10] explained that elderly patients who visit the emergency room often have non-specific complaints due to numerous factors including comorbidities, cognitive and functional impairment and communication problems. This could similarly be an issue during the dispatching process at the EMDC. It has been shown that cardiac arrest, which frequently occurs in the elderly [11], is a medical condition that is difficult to spot [12].

### EMS vehicle cancellation triaged with the UPC

The data demonstrated that 34.7% of the dispatched EMS vehicles after triage with the UPC end up being cancelled. This number is higher than the amount cancelled for non-UPC cases, which is 29.7%. This study’s scope did not allow for the determination of under and over triage.

Nonetheless, the results could indicate that, in general, over-triage might be an issue in Copenhagen, and particularly when regarding the UPC, as it might suggest that in the first instance, more or higher-level emergency vehicles are sent out than necessary. However, it could also denote the opposite – if more cars are cancelled, it might mean that cases are more often under-triaged as vehicles could be cancelled in instances where they were essential.

A study conducted in Vaud, Switzerland, evaluated over and under triage from their criteria-based dispatch system. They found that, both in cases of under and over triage, *"undefined problem"*, was the most used criterion. This criterion represented 38% of over triage and 83.6% of under triage cases [13]. Considering the same dispatch system was used as in the EMDC, this could indicate that the UPC indeed leads to more over and under triage.

Møller et al. [5] found a higher mortality rate for emergency priority level B cases categorized as UPC. Level B cases are cases that are urgent, but not life threatening. Møller et al. state that this might imply that a higher priority level should have been used in the medical dispatch process. In other words, that there was a problem of under-triaging, with a subsequent detrimental effect on patient outcomes. However, they stated that if a higher priority level is used in the medical dispatch process, this will increase EMS demand whilst there are limited EMS resources. Therefore, it could interfere with adequately responding to other patients in need of EMS services [5].

### The impacts of sensitizing medical dispatchers about the use of the UPC to reduce its use

As can be seen from the register-based results, the use of the UPC has been steadily decreasing over the period of the study. Throughout the period of the study, medical dispatchers have been alerted about the implications of using the UPC with the attempt to reduce its use. The use of the UPC has shown the greatest decrease out of all the different categories. This could indicate that alerting medical dispatchers about the implications of the use of the UPC at the EMDC in Copenhagen had a positive effect.

Other studies have also shown positive effects after the implementation of a new protocol in EMDCs. The introduction of a new protocol in EMDCs to improve cardiac arrest identification by medical dispatchers and increase conduction of medical-dispatcher-assisted CPR to patients has shown to be effective [14, 15]^.^ Although these studies were not related to the UPC, it further exemplifies that new protocols can have beneficial effects in the medical dispatching process, similarly to how the protocols implemented at the EMDC in Copenhagen have reduced the usage of the UPC.

### Implications

This study shows that the median age of patients dispatched with the UPC is higher than that of patients dispatched with symptom-specific categories. It demonstrates that UPC incidents are more often cancelled, and therefore could have negative effects on patient outcomes or the efficient use of resources. Moreover, this study reports a reduction in the use of the UPC after educating medical dispatchers with the aim to improve the triage process and reduce the use of the UPC. This research sheds light on research opportunities and improvements with regard to UPC use.

### Limitations

A limitation of the study is that although observations can be drawn from the results, no causal inferences can be established, and further research is required to achieve that. Qualitative research could indicate why medical dispatchers decided to opt for the UPC, their opinion about the use and existence of this category, and this could show where improvements could be made in the EMDC. Moreover, suppose the patient outcomes could be linked to the data. In that case, it could clarify whether the triage is not excessive or rather insufficient and measures could subsequently be taken to provide a more accurate medical dispatching process.

Moreover, it is known that the dispatchers were alerted about the use of the UPC, yet there is no further information about how this process was undertaken. The decrease of the UPC could be related to educating medical dispatchers about the implications of using the UPC, yet this cannot be proven.

Additionally, the analysis does not include the medical dispatchers' factors, such as sociodemographic factors, their professional background, or mental state, which might affect emergency call categorization.

### Further research

This research showed that the patient and dispatch characteristics of the UPC are different than the patient and dispatch characteristics of symptom-specific categories. To better investigate the impacts of the UPC, qualitative research and research on patient outcomes need to be done.

Moreover, if it is known how the medical dispatchers were alerted about the implications of the use of the UPC, then this could facilitate implementation of future improvements in medical dispatching, not only at the EMDC but also in other Danish regions or even internationally. Another topic that could be interesting to regard for future research is how artificial intelligence could aid medical dispatchers in choosing a correct medical dispatching category and thereby reduce their use of the unclear category. Blomberg et al. [16, 17] emphasize artificial intelligence's skill to recognize out of hospital cardiac arrest, yet acknowledge that future studies are needed to improve human–computer interaction.

Although this research has denoted some possible gaps in the system that could be improved to reduce the use of the UPC, it would be interesting to look into other tools to reduce its use. This study could lead to further research and serve as a starting point for an improved, more efficient EMDC in Copenhagen. A better EMDC could subsequently benefit Danish society. Furthermore, other EMDCs in Europe could take note of this research. This report could be a stimulus for EMDC leaders to investigate their medical dispatching categorization system and the implications that come along with it, to make further improvements.

## Conclusions

The UPC has different dispatch characteristics than the symptom-specific categories, with potential negative effects on the medical dispatch process. Moreover, it was found that the median age of individuals dispatched with the UPC is higher than those dispatched with symptom-specific categories. Nonetheless, the UPC has been decreasing over time after the medical dispatchers were alerted about the implications of using the UPC. Notably, this research has illustrated which aspects of the UPC need further research to make future improvements.

## Data Availability

Data available from corresponding author upon reasonable request.

## Abbreviations

EMS: Emergency medical services
EMDC: Emergency medical dispatch center
CBD: Criteria based emergency dispatch system
UPC: Unclear problem category
